# A Cell-Based Method for Screening RNA-Protein Interactions: Identification of Constitutive Transport Element-Interacting Proteins

**DOI:** 10.1371/journal.pone.0048194

**Published:** 2012-10-25

**Authors:** Robert L. Nakamura, Stephen G. Landt, Emily Mai, Jemiel Nejim, Lily Chen, Alan D. Frankel

**Affiliations:** 1 Department of Biochemistry and Biophysics, University of California San Francisco, San Francisco, California, United States of America; 2 Department of Biology, San Francisco State University, San Francisco, California, United States of America; International Centre for Genetic Engineering and Biotechnology, Italy

## Abstract

We have developed a mammalian cell-based screening platform to identify proteins that assemble into RNA-protein complexes. Based on Tat-mediated activation of the HIV LTR, proteins that interact with an RNA target elicit expression of a GFP reporter and are captured by fluorescence activated cell sorting. This “Tat-hybrid” screening platform was used to identify proteins that interact with the Mason Pfizer monkey virus (MPMV) constitutive transport element (CTE), a structured RNA hairpin that mediates the transport of unspliced viral mRNAs from the nucleus to the cytoplasm. Several hnRNP-like proteins, including hnRNP A1, were identified and shown to interact with the CTE with selectivity in the reporter system comparable to Tap, a known CTE-binding protein. *In vitro* gel shift and pull-down assays showed that hnRNP A1 is able to form a complex with the CTE and Tap and that the RGG domain of hnRNP A1 mediates binding to Tap. These results suggest that hnRNP-like proteins may be part of larger export-competent RNA-protein complexes and that the RGG domains of these proteins play an important role in directing these binding events. The results also demonstrate the utility of the screening platform for identifying and characterizing new components of RNA-protein complexes.

## Introduction

Mapping the networks of biological interactions is one of the major challenges of the post-genomic era. For RNA-protein complexes, many of the important interactions occur in the context of multi-protein ribonucleoprotein (RNP) assemblies, making it desirable to understand how individual interactions fit within these larger molecular frameworks. A number of techniques have been developed to characterize RNA-binding interactions, including direct binding methods such as gel shift, and fluorescence anisotropy assays, RNA mapping methods such as nuclease mapping, chemical probing, cross-linking and immunoprecipitation (CLIP), cross-linking and analysis of cDNAs (CRAC), selective 2′-hydroxyl acylation analyzed by primer extension (SHAPE), and nucleotide analog interference mapping (NAIM), genetic methods, such as the yeast 3-hybrid assay, microarray-based methods, and mass spectrometry methods [1]–[5] (reviewed in [6], [7]). Each method has inherent strengths and limitations but collectively they provide the complementary data needed to uncover meaningful biological interactions.

We have developed a method to identify proteins that interact with RNA or assemble into larger RNA-protein complexes in the context of mammalian cells. The method is based on transcription activation of the HIV LTR, where an interaction between the HIV Tat protein and the TAR RNA element located at the 5′-end of nascent transcripts enhances elongation [8]. The system is modular in that an RNA target of interest can be cloned in place of TAR, and an interacting protein can be fused to Tat to activate expression of a reporter gene. This ‘Tat-hybrid assay’ previously has been used to characterize several heterologous RNA-protein interactions, including the Rev-RRE, SF1-U2AF65-branchpoint, and U1A-U1 snRNA interactions [8]–[10]. The method has two key advantages. First, the RNA-protein complex is presented in a mammalian cellular environment where required accessory factors, chaperones, or post-translational modifying enzymes are available to assemble proper complexes. Second, the assay has high sensitivity and is able to detect both direct RNA-binding proteins as well as those that assemble into complexes via protein-protein interactions and may not directly contact the RNA. To adapt the system into a robust screening platform, we developed several new reagents and overcame some key technical issues associated with identifying clones from mammalian cells. We created a cDNA library fused to Tat, optimized a protoplast fusion method to clonally introduce the library plasmids into mammalian reporter cells, and developed procedures to sort positive clones based on GFP intensities and efficiently recover plasmid DNA in a large-scale screening format.

**Figure 1:**
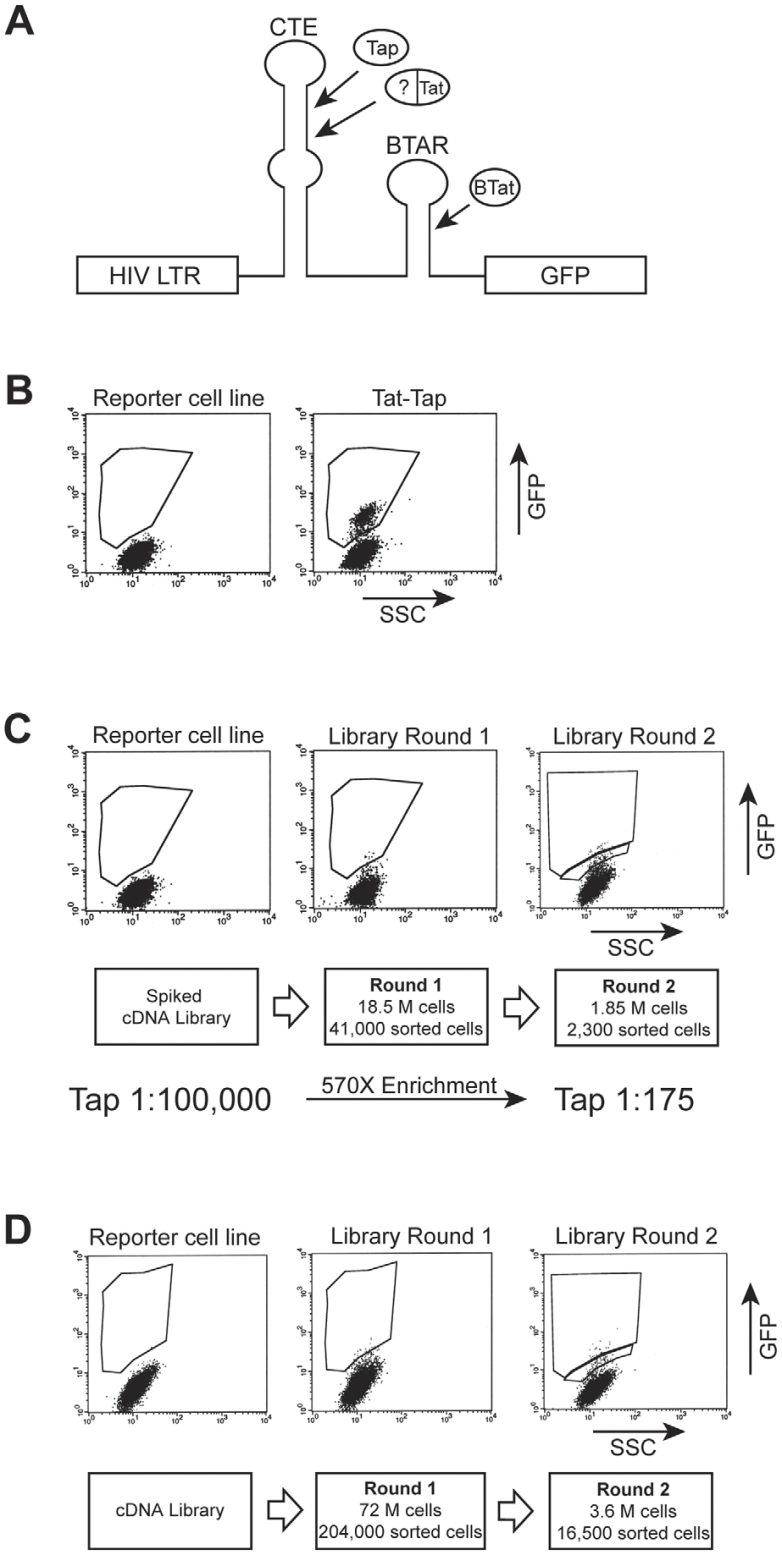
Schematic diagram of the Tat-hybrid cDNA library screen. (A) Transcription elongation from the HIV LTR GFP reporter plasmid is enhanced only when a Tat-fusion protein interacts with the CTE RNA target, which is located in place of TAR at the 5′ end of the mRNA. The CTE reporter was integrated into HeLa cells to obtain a consistent, highly responsive cell line. The BIV TAR hairpin binds BIV Tat and serves as a positive control throughout the library screen and subsequent analyses of clones. (B) GFP activation level observed with a Tat-Tap positive control. SSC represents side scatter. (C) An HIV Tat-cDNA fusion library spiked with the Tap positive control (at 1∶100,000) was clonally introduced into the reporter cell line by protoplast fusion. CTE interacting clones were identified by FACS, plasmid DNA was isolated and re-introduced into *E. coli*, and a second round of library screening was carried out. Plasmids recovered after the second round of screening were identified as Tat-Tap clones by PCR. (D) cDNA library screen as in (C) except without the Tat-Tap positive control. Plasmids recovered after the second round sort were sequenced and further characterized.

As a test of the method, we wished to apply the screen to a virus-host interaction, as studies of viral RNA-protein complexes often shed light on underlying cellular processes. For example, studies of the HIV Rev-RRE (Rev response element) RNA interaction led to the discovery of the Crm1-mediated export pathway, which transports the HIV and some cellular RNAs from the nucleus to the cytoplasm [11]–[14]. To export HIV RNAs, Rev binds to the RRE located in an intron of the *env* gene and interacts with Crm1 via a leucine-rich nuclear export sequence (NES). This allows export of the partially spliced and unspliced viral mRNAs, which are translated into the viral structural proteins and provide genomic RNA for packaging.

**Figure 2:**
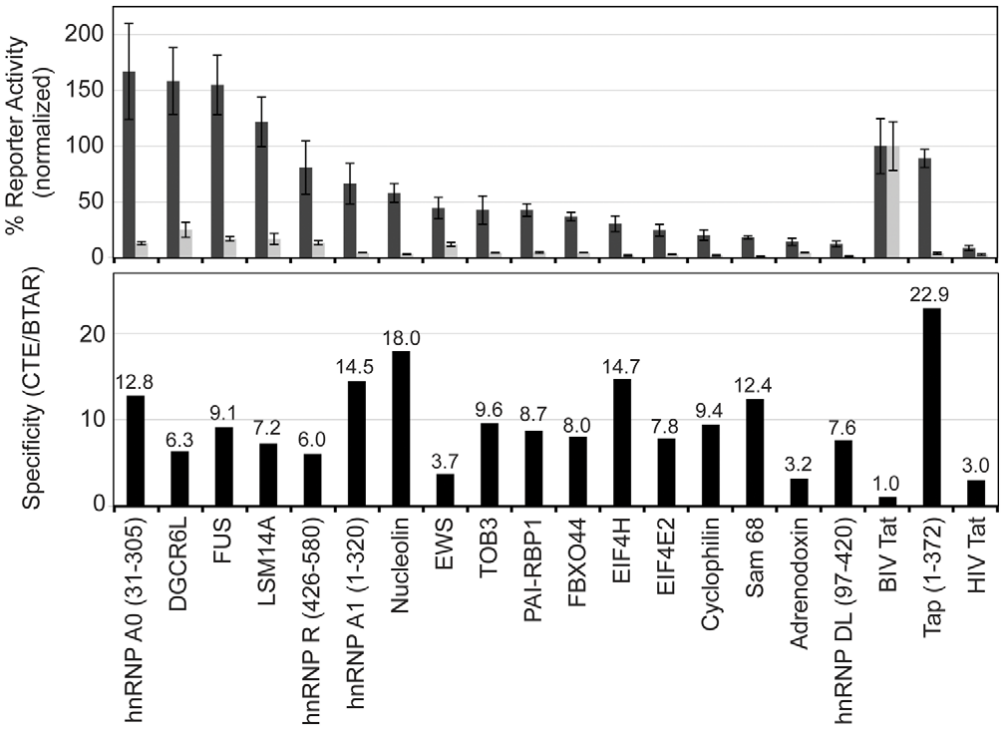
*In vivo* binding of CTE-interacting clones. (Top) Activation assays with individual clones were performed on pHIV LTR CTE BTAR CAT (dark grey bars) and pHIV LTR BTAR CAT (light grey bars) reporters. Each clone was co-transfected along with the reporter DNA into HeLa cells and CAT activity was measured after 48 h. Plasmids encoding BIV Tat and Tap were included as positive controls. HIV Tat, a negative control, displayed a 2 to 3-fold preference for the CTE reporter, and thus library clones with specificities in that range were considered non-specific. To ensure that the expression of the library clones was consistent, each was independently co-transfected into HeLa cells with a pHIV LTR TAR-CAT reporter and CAT activity was found to be similar between all clones (data not shown). Library clones can activate transcription via HIV TAR because the fusions are to full-length Tat, which is able to bind HIV TAR. (Bottom) CTE specificity of each clone represents the ratio of activities on the CTE BTAR reporter to the BTAR reporter. Activities were normalized to 1 for BIV Tat specificity. The positive control Tat-Tap (1–372) fusion conferred the highest specificity for the CTE but other clones, including hnRNP A1, displayed similar levels of specificity.

We targeted the current screen to a different RNA export element, the constitutive transport element (CTE) from Mason Pfizer monkey virus (MPMV). This simple retrovirus does not encode a regulatory protein like Rev but rather uses the CTE RNA structure to export unspliced viral mRNAs by interacting directly with cellular proteins [15]. The CTE does not mediate export via Crm1, as saturating amounts of CTE RNA microinjected into Xenopus oocytes inhibit CTE-dependent and mRNA export but not Rev-mediated export [16], [17]. CTE-mediated export also is insensitive to leptomycin B, a specific inhibitor of the Crm1 pathway [18], highlighting the existence of distinct export pathways. Because the CTE competes with cellular mRNA export, it was anticipated that CTE-interacting proteins also might be used in cellular export pathways [16], [17]. Indeed, Tap, the major export receptor for cellular mRNAs, was first found as a CTE-binding protein [19]. Tap binds directly to the CTE with high affinity and specificity [19], but also binds non-CTE-containing mRNAs with lower affinity [20]–[22].

**Table 1:** CTE-interacting clones identified by the cDNA library screen.

Clone	Insert length (Amino Acids)	No. of isolates	RNA-binding	No. of RGGs	Comments	Ref.
hnRNP A0	31–305, 122–305	2	Yes	4	2xRRM member of the hnRNP family that interacts with pre- mRNAs and has multiple roles in RNA processing and nuclear export.	[54]–[56]
DGCR6L	1–220	1	no	0	DiGeorge syndrome critical region gene 6-like protein that shares homology with Drosophila gonadal (gdl) protein and human laminin gamma-1 chain.	[57], [58]
FUS/TLS	187–526	1	Yes	19	Involved with multiple activities related to RNA transcription, splicing, metabolism, and export.	[59]
LSM14A/RAP55	308–463	1	Yes	8	Member of the LSm family that localizes to stress granules under stress conditions and may play a role in mRNA sequestration.	[60]
hnRNP R	452–633,480–633	2	Yes	6, 7	3xRRM member of the hnRNP family that interacts with pre- mRNAs and has multiple roles in RNA processing and nuclear export.	[55], [61]
hnRNP A1	1–320, 159–320, 144–320	21	Yes	4	2xRRM member of the hnRNP family that interacts with pre- mRNAs and has multiple roles in RNA processing and nuclear export.	[55], [62]
Nucleolin	519–710	1	Yes	4	Implicated in many aspects of RNA biology including ribosomal RNA transcription and processing, ribosome assembly, nucleosome remodeling, and nuclear export.	[38], [39]
EWS	554–656	1	Yes	12	Involved with multiple activities related to RNA transcription, splicing, metabolism, and nuclear export.	[59]
TOB3/ATAD3p	107–285	1	no	0	DNA-binding protein present in mitochondrial nucleoids.	[63]
PAI-RBP1/SERPINE/CGI-55	1–408	1	Yes	7	mRNA destabilizing factor that binds the PAI-1 mRNA and enhances its degradation; also implicated with stress granules through association with the RNA-binding protein ORF1p.	[64], [65]
FBXO44/Fbx30/FBG3/FBXO6a	80–224	1	no	0	Member of the F-box family, components of the ubiquitin protein ligase complex SCF (Skp1/Cdc53-Cullin1/F-box).	[66], [67]
EIF4H	1–235	1	Yes	1	Modulates the activity of the eIF4A RNA helicase, promoting unwinding of the 5′ UTR and facilitating binding to the ribosome.	[68], [69]
EIF4E2/4E-LP/4EHP/EIF4EL3/IF4e	1–245	1	Yes	1	Member of the eukaryotic initiation factor 4E (eIF4E) family of Cap-binding proteins.	[70]
Cyclophiln A	1–166	4	no	0	Member of the peptidyl-prolyl cis-trans isomerase (PPIase) family that binds cyclosporin A and is involved in cyclosporin A-mediated immunosuppression.	[71]
Sam68	1–443	1	Yes	2	Member of the STAR (Signal transduction and activation of RNA) family that was reported to enhance the activity RRE- and CTE-dependent reporters through a synergistic interaction with Tap.	[36], [37], [72]
Adrenodoxin	1–184	1	no	0	Iron-sulphur protein that serves as an intermediate during electron transfer from NADPH to P450.	[73]
hnRNP DL	97–420	1	Yes	2	2xRRM member of the hnRNP family that interacts with pre-mRNAs and has multiple roles in RNA processing and nuclear export.	[54], [55]

Here we present the development of the Tat-hybrid screening platform and the results of an initial CTE library screen. We provide evidence that hnRNP A1 assembles into CTE complexes, in part by interacting with Tap and RNA. The interaction requires the RGG domain of hnRNP A1, a common domain in RNA-binding proteins that often contains methylated arginines [23], [24]. We find that other proteins and peptides that contain RGG domains also interact with Tap and the CTE, suggesting that the formation of such RNP complexes may function in viral and cellular mRNA export pathways.

**Figure 3:**
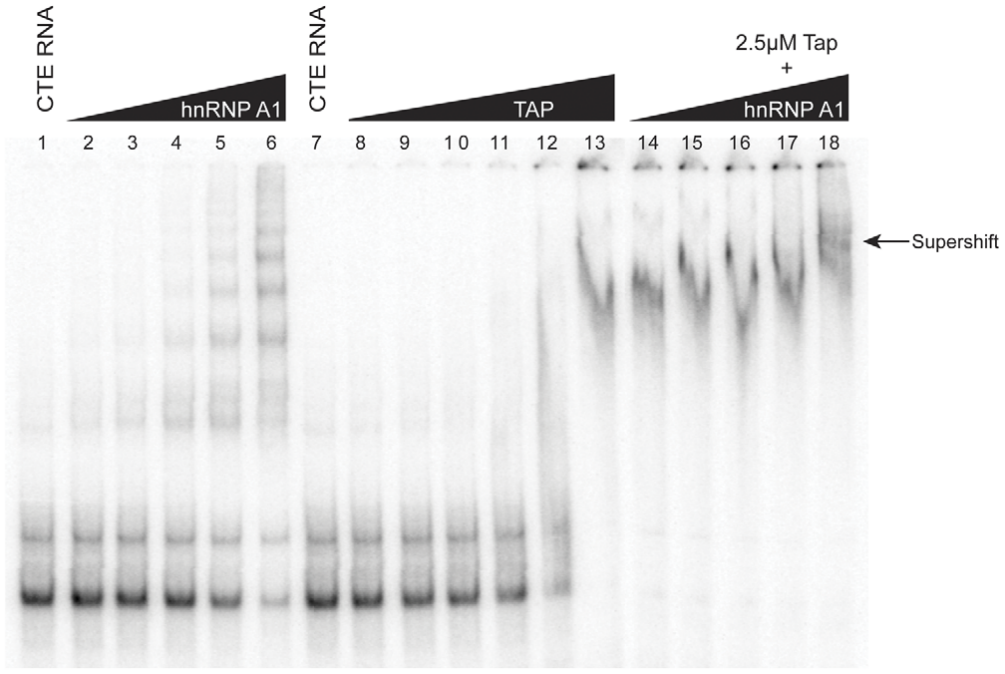
*In vitro* binding of the CTE, hnRNP A1, and Tap. Gel shift experiments were performed with *in vitro* transcribed [^32^P]-labelled CTE, GST-hnRNP A1, and/or GST-Tap in the binding reactions. Lanes 2–6 show a titration of GST-hnRNP A1 (0.045, 0.090, 0.18, 0.36, and 0.71 µM) and lanes 8–13 show a titration of GST-Tap (0.075, 0.15, 0.31, 0.63, 1.25, and 2.5 µM). In lanes 14–18, CTE complexes with 2.5 µM GST-Tap were titrated with hnRNP A1, resulting in a supershifted complex at the highest concentration (lane 18).

## Materials and Methods

### Plasmids

RNA reporter plasmids were constructed using the pHIV LTR vector [25]. The MPMV CTE, corresponding to nucleotides 7388–7549 (Genbank Accession #NC_001550 and kindly provided by David Rekosh) was PCR amplified, introducing an AflII site at the 5′ end and an NheI site followed by a 28-nucleotide BIV TAR hairpin [26] and an SpeI site at the 3′end, and cloned to create pHIV LTR CTE BTAR GFP. A corresponding CAT reporter was created by PCR amplifying a fragment with BstXI and NotI ends and cloning in place of GFP. The pHIV LTR CTE BTAR vectors contain the CTE followed by the BTAR 28-nucleotide hairpin with an additional lower stem of HIV-1 TAR.

**Figure 4:**
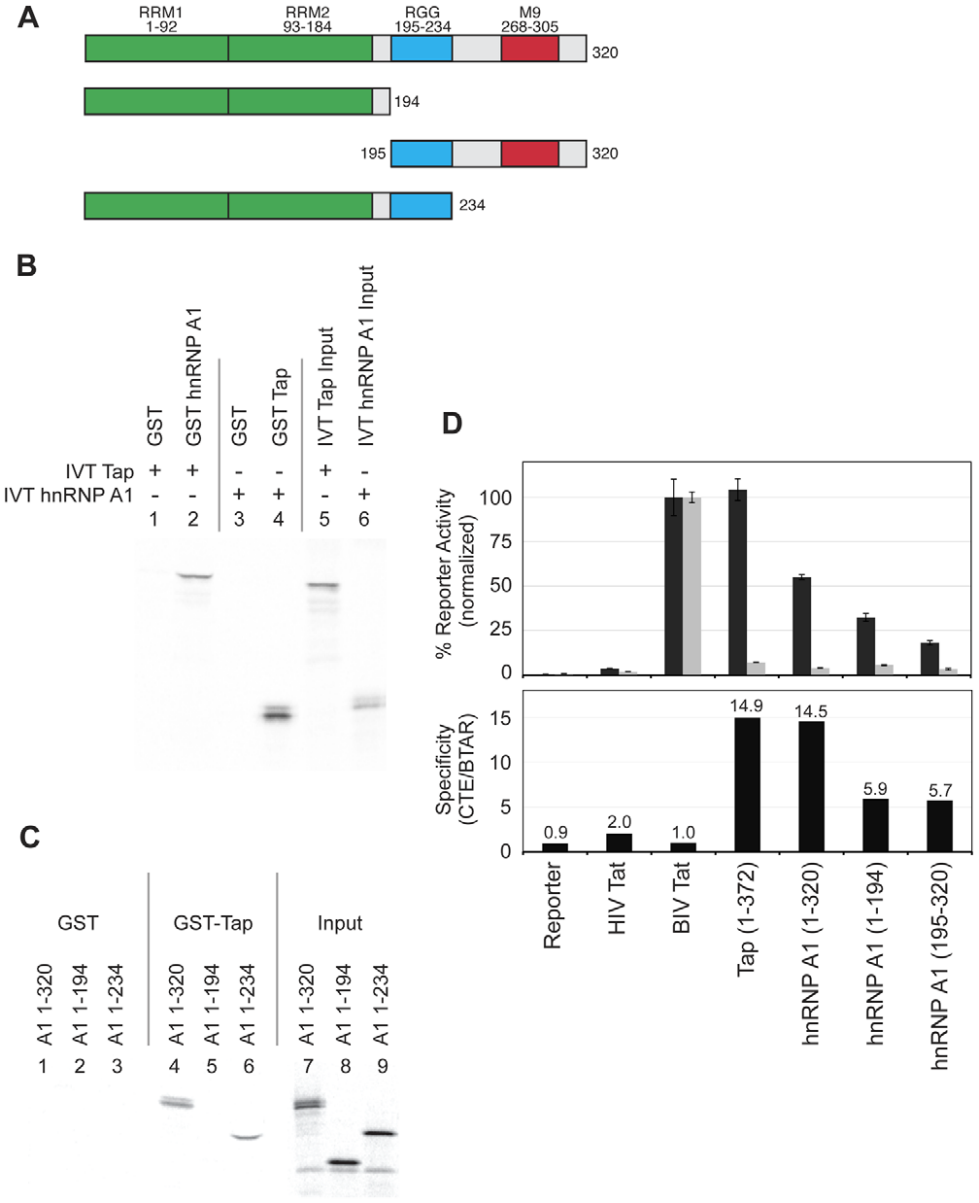
Mapping the interaction domains of hnRNP A1. (A) Schematic diagram of the hnRNP A1 constructs (1–320, 1–194, 195–320, and 1–234) used for *in vitro* pull down or Tat-hybrid reporter experiments. (B) GST pull-down assays with hnRNP A1 and Tap. This experiment utilized GST-hnRNP A1 (1–320) or GST-Tap (1–619) and the corresponding [^35^S]-labeled *in vitro* translated (IVT) proteins as indicated. The two input lanes (5 and 6) represent 1/10 of the *in vitro* translated proteins used for binding. GST alone was used as a negative binding control. (C) GST-Tap pull-down assays of full-length hnRNP A1 and two deletion mutants (1–194 and 1–234). The three input lanes (7–9) represent 1/10 of the *in vitro* translated proteins used for binding. GST alone was used as a negative binding control. (D) Activity and specificity of the hnRNP A1 truncations in the Tat-hybrid assay. Activities were determined using the same methods as in Fig. 2. Tap 1–372 and BIV Tat were included as positive controls and HIV Tat as a negative control.

To express HIV Tat fusion proteins, we generated a mammalian codon-optimized pSV2-Tat vector with a NotI site (encoding Gly-Gly-Arg following Tat amino acid 72) at the 3′ end of the gene by cloning a synthetic HindIII-XhoI fragment into pSV2 Tat [26], [27]. We inserted an additional 2 kB stuffer fragment to facilitate purification of the doubly-digested NotI-XhoI vector for fusion protein cloning [25]. Amino acids 2–372 from a TAP cDNA (kindly provided by E. Izzuaralde), as well as hnRNP A1 variants with amino acids 1–194 or 195–320 or mutants using cDNA templates, were PCR amplified and cloned as in-frame fusions into the codon-optimized vector. Clones to express GST fusion proteins were generated by inserting PCR fragments encoding Tap 1–619 or hnRNP A1 1–320 into BamHI and EcoRI sites of pGEX2T. Plasmids for *in vitro* translation were cloned into BamHI and HindIII or AflII and BamHI sites in pcDNA3.1+ (Invitrogen).

**Table 2:** Properties of the out-of-frame library clones.

Clone	Insert Length (amino acids)	Number of RGs	Number of RGGs
HIV Tat 1–72	-	0	0
Tat-N3	138	8	0
Tat-N12	177	1	1
Tat-N26	118	5	4
**Purified Protein**			
GST	-	1	0
GST-N3	152	8	0
GST-N12	189	1	1
GST-N26	43	2	3
6X His hnRNP A1	N/A	5	4

### Cell lines

A HeLa cell line expressing the pcDNA3 HIV-1 LTR-CTE-BTAR-GFP reporter was generated to obtain a consistent background for library screens. The plasmid was tranfected into HeLa cells using Lipofectin (Invitrogen) and stable integrants were selected using neomycin (G418) (800 µg/ml) for 10 days. Resistant cells were transfected with the pSV2 BIV Tat plasmid to activate the GFP reporter and GFP-expressing cells were isolated by FACS into 96-well plates. Stable cell lines were evaluated by transfecting with pSV2 BIV Tat plasmid and monitoring GFP signal-to-noise by FACS scans to identify those with highest activation levels.

**Figure 5:**
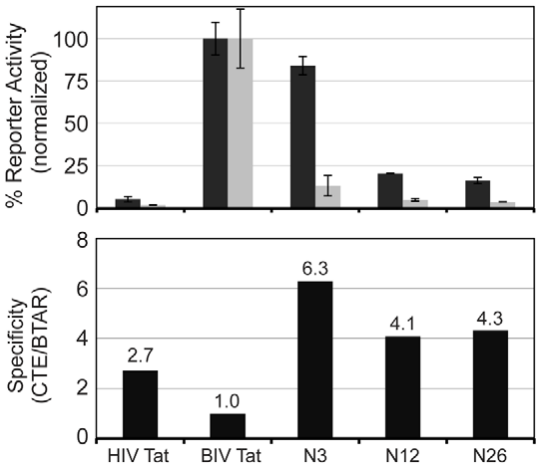
Analyses of non-coding library clones. Activities and specificities of the out-of-frame clones in the Tat-hybrid assay were determined as in Fig. 2. All fusions were independently tested using the HIV TAR CAT reporter to confirm that expression levels and transfection efficiencies were similar (data not shown).

### cDNA library

PolyA+ mRNA prepared from HeLa cells grown in spinner flasks was reverse transcribed using a poly(T)_18_ primer with an XhoI site at the 5′end and BRL SuperscriptII reverse transcriptase according to manufacturers instructions in the presence of 10 mM 5-methyl dCTP. Second strand synthesis was carried out using *E. coli* DNA ligase, *E. coli* DNA pol I, and RNase H according to manufacturer instructions. cDNA was size-fractionated on a 5–20% potassium acetate gradient. NotI adapters (5′GGCCGCGCTCTCAGTG, 5′CACTGAGAGCGC) were ligated to double-stranded cDNA, and cDNA was digested with XhoI and cloned into pSV2Tat containing the stuffer. 6.7×10^6^ independent library clones were obtained and >75% had an insert of average size ∼1.8 kB.

### Library screening

Protoplast fusion was carried out as previously described [8]. Briefly, the cDNA library was transformed into *E. coli* DH-5α and grown to an O.D. 600 of 1.0 at 37°C in LB media containing 100 µg/ml ampicillin. The plasmids were then amplified overnight with 250 µg/ml chloramphenicol. Protoplasts were prepared as described [28] and diluted into DMEM containing 10% sucrose and 10 mM MgCl_2_. CTE HeLa cells were grown in 75 cc flasks and washed with serum free DMEM. A protoplast suspension was added to the flask and centrifuged at 1,650 g for 10 minutes at room temperature. The supernatant was removed and 4 ml of 50% (w/v) PEG 1500 diluted in DMEM was added. After 2 minutes, the supernatant was removed and cells were washed twice with serum-free DMEM. The cells were resuspended in DMEM containing 10% fetal bovine serum and penicillin/streptomycin (100 Units/100 µg/ml), and incubated for 48 h.

### Fluorescence activated cell sorting (FACS)

Protoplast-fused cells were harvested with cell dissociation buffer (CDB, Invitrogen), washed with PBS, and resuspended in PBS containing 5% CDB, 0.3% fetal bovine serum, and 1 µg/ml propidium iodide. In the initial round of cell sorting, a sorting window was drawn manually to include approximately 0.1% background cells. Subsequent sorting windows were drawn to enrich for more highly expressing GFP-positive cells. Samples were analyzed using a FACScan flow cytometer (Becton Dickinson) or sorted using a FACSvantage (Becton Dickinson) cell sorter. Cells were excited at 488 nm and a 530+/−10 nm band pass filter was used to detect GFP emission. Flow cytometry was performed at the Howard Hughes Medical Institute (University of California, San Francisco) and FACS data were analyzed using CELLQuest softward (Becton Dickinson).

### Plasmid recovery

Sorted cells were mixed with 10–20,000 carrier cells, spun down, and resuspended in 10 µl of TE buffer (10 mM Tris, pH 8.0, 1 mM EDTA) containing 0.2 mg/ml tRNA. Plasmids were prepared by alkaline lysis [29]. Electrocompetent cells were prepared by growing 500 ml cultures of *E. coli* DH-5α to O.D. 600 of 0.7, centrifuging to remove the media, and washing with 2 L of ice cold water through a 0.22 µM sterile filter. Cells were resuspended in 10% glycerol, and 50 µl aliquots were used for electroporation. High efficiencies of transformation were achieved using these methods (approximately 5×10^11^ colonies/µg of DNA).

### Transfections, CAT assays, and FACS scans

HeLa cells or the HeLa CTE cell line were cultured in DMEM (Gibco-BRL) with 10% fetal bovine serum, penicillin/streptomycin, and grown in 12-well plates for transfection experiments. For CAT assays, 50 ng of reporter plasmid (pHIV-LTR-CTE-BTAR-CAT, pHIV-LTR-BTAR-CAT, and pHIV-LTR-CAT) and 5–25 ng of Tat-fusion plasmid (pSV2tat72-based) were mixed with carrier Bluescript plasmid (adjusted to 1 µg total DNA) in Optimem low serum medium (Gibco-BRL). DNA was transfected into cells using Lipofectin (Gibco-BRL). Cells were grown for 48 h post-transfection, harvested, and subject to CAT assays as described previously [26]. CAT activity from different reporters was normalized to the activity of BIV Tat when appropriate. Library clones were tested on the pHIV-LTR-CAT reporter to control for transfection efficiency. For FACS scans, CTE cells were transfected with the library clone, or protoplast fused with *E. coli* containing the library plasmid, and analyzed by FACS scan after 48 h.

### Protein purification and gel shift assays

*E. coli* cells (strain BL21) expressing GST-fusion proteins were inoculated in LB-Amp and grown to O.D. 600 of 0.7–1.0. Plasmids were induced with 100 µM IPTG for 6–8 hours of induction with 100 µg/ml IPTG, then centrifuged, resuspended in column buffer (50 mM Tris, pH 8.0/150 mM NaCl/2 mM DTT/10% glycerol/1 mM PMSF/0.5% Triton X-100) and sonicated. The lysate was centrifuged at 15,000 g and the supernatant was applied to a column containing 1 ml glutathione agarose. The beads were washed with several volumes of column buffer, and the GST proteins were eluted with 5 mM glutathione in column buffer. For RNA transcription, the CTE was PCR amplified and cloned into the SacI and KpnI sites of a pBluescript SK vector. ^32^P labelled RNA for gel shifts was transcribed using T7 polymerase and purified by SDS-PAGE. Binding reactions for gel shift assays were carried out at 4°C and contained 10 mM HEPES/50 mM KCl/2.5 mM MgCl_2_/500 µM DTT/10% glycerol/0.025% NP40 and 1000–5000 cpm RNA, 0.02 mg/ml Poly (C) RNA, GST-fusion protein, and RNAsin (Promega). After binding, 0.02 mg/ml heparin was added and samples were run on 6% acrylamide Tris-Glycine native gels and analyzed using Phosphorimager software.

### GST pull-down assays

*E. coli* cells (strain BL21) expressing GST-fusion proteins were inoculated in LB-Amp and grown to O.D. 600 of 0.7–1.0. Plasmids were induced with 100 µM IPTG and grown for 2–8 h at 30°C. Cells were centrifuged to remove the media, and resuspended in lysis buffer (20 mM Tris-HCl pH 8.0/200 mM NaCl/1 mM EDTA/0.5% NP40) with 25 µg/ml PMSF and 0.2% protease inhibitor cocktail (Sigma) [30]. The cell suspension was sonicated, centrifuged at 16,000 g, and the supernatant transferred to a fresh tube. Glutathione agarose beads (Sigma) were prepared by swelling in lysis buffer, and 50 µl of cell lysate was mixed with 50 µl of bead slurry and 2 µl of *in vitro* translated protein in each pull-down reaction. *In vitro* translated proteins were prepared using TnT T7 Quick Coupled Transcription/Translation System (Promega) according to the manufacturer's protocol with [^35^S]-Methionine (Amersham). GST binding reactions were incubated at 4°C for 1 h. After binding, the beads were centrifuged at 16,000 g and the supernatant was removed. Beads were washed 4 times with lysis buffer, and resuspended in SDS-PAGE loading buffer. Samples were run on 10% SDS-PAGE gels and analyzed by Phosphorimager (Molecular Dynamics) using Imagequant software (Molecular Dynamics).

### *In vitro* methylation

Methylation assays were performed essentially as described [31]. A plasmid encoding GST-Prmt1 was a kind gift of H. Herschman. 23 µl reactions containing 4 µM GST-fusion protein, 3 µl [^3^H]-Ado-Met (NEN Life Science, 0.55 mCi/ml), and 0.2 µg of GST-Prmt1 were incubated at 37°C for 1.5 h. The reactions were mixed with 7 µl of SDS sample buffer and samples were run on a 10% SDS-PAGE gel, transferred to a nitrocellulose filter, and analyzed using Phosphorimager and Imagequant software.

## Results

### A library screen to identify CTE-interacting proteins

We have developed a screening platform for identifying proteins that interact with RNA in the context of mammalian cell lines. The platform makes use of Tat-mediated HIV transcription activation assays [8], [25] in which HIV-1 Tat enhances elongation from the HIV-1 LTR by binding to the TAR RNA site located at the 5′ end of the nascent viral mRNAs. For this screen, TAR was replaced by the CTE (Fig. 1A), and a Tat-fusion library was generated with cDNAs fused to HIV-1 Tat, expecting that fused CTE-binding proteins would bind the RNA and activate GFP expression. The GFP reporter also encoded the BIV TAR element downstream of the CTE, providing an independent binding site for BIV Tat, but not HIV Tat [26]. In practice, BIV TAR served three critical roles: first, as a positive control for reporter activity; second to quantify the efficiency of protoplast fusion delivery; and third to allow use of the BIV Tat-TAR interaction as a calibration standard to score the specificities of positive library clones. The reporter plasmid was stably integrated into HeLa cells and a cell line was selected that displayed low constitutive GFP expression but high expression when transfected with BIV Tat.

We constructed a cDNA library derived from HeLa cells that contained an average insert of ∼1.8 kb fused to amino acids 1–72 of Tat. This portion of Tat encodes both the activation domain (AD, amino acids 1–48) and arginine-rich motif (ARM) RNA-binding domain (amino acids 49–57). The ARM was included because it also serves as an NLS [32], [33] and helps generate higher GFP signals, particularly for weak RNA binding proteins, while not appreciably increasing activation levels through nonspecific RNA binding (see Figure 2, HIV Tat). Because Tat 1–72 fusions also bind HIV TAR, we derived the additional benefit of conducting parallel assays with an HIV TAR reporter to quantify functional expression of each fusion protein [10]. The library was delivered into HeLa reporter cells by protoplast fusion such that, on average, less than one bacterial cell is fused to each HeLa cell and thus delivery of a single library plasmid is nearly clonal [8], [34]. Protoplast delivery also has the advantage that GFP expression levels are relatively consistent among the fused cell population, as demonstrated by FACS analyses with the CTE-binding protein, Tap (amino acids 1–372), fused to Tat (Fig. 1B). Transient transfection, by contrast, generates a more heterogeneous population [8] and makes the choice of a sorting window for screening somewhat more arbitrary. Furthermore, recovering plasmids from GFP-positive cells sorted by FACS is more efficient following protoplast fusion, as many copies of a single plasmid are delivered per HeLa cell and can be captured by electroporation of highly competent *E. coli* cells [8].

To calibrate a FACS sorting window to identify positive clones and to determine an enrichment factor per round of plasmid delivery, FACS, and recovery, we conducted a mock screen by adding Tat-fused Tap (1–372) into the cDNA library at a 1∶100,000 ratio and performing two rounds of screening (Fig. 1C). In round one, we analyzed ∼18.5×10^6^ HeLa cells and sorted ∼41,000 GFP positive cells using a conservative window derived from the activity of Tat-fused Tap (Fig. 1C, center panel). Following electroporation into *E. coli*, we recovered 20,500 transformants and generated a plasmid pool. In a second round we used a higher sorting window to enrich for strong GFP activators (Fig. 1C, right panel), analyzed 1.85×10^6^ HeLa cells, and recovered 2300 GFP-positive cells and 900 transformants. PCR analyses of 700 transformants identified four Tap clones, representing an enrichment factor of 570 over the two rounds.

We next conducted the library screen targeting the CTE, beginning with 72×10^6^ HeLa cells and resulting in 16,500 sorted cells and 5,000 transformants after the two rounds (Fig. 1D). We conducted a third round which yielded a higher fraction of positives but chose to analyze individual clones following the second round to preserve diversity and include potentially weaker CTE-binding proteins. DNA was prepared from 1,500 clones and digested with restriction enzymes to reveal the size of the insert. About half of the clones had small or no inserts or abnormalities in the plasmid backbone and were discarded. The several hundred remaining clones were retested individually for activity on the CTE reporter and those showing moderate to high GFP levels were sequenced.

### Identities and activities of CTE-interacting clones

To more quantitatively evaluate the activities of the positive Tat-fused clones, we measured the activation levels of the 17 most active clones utilizing a CTE CAT reporter instead of GFP (Fig. 2, Top). In addition, we tested the clones on an unrelated BIV TAR reporter to assess the specificity of each clone for the CTE (Fig. 2, Bottom). Figure 2 shows that several of the clones conferred strong activity on the CTE reporter and low activity on the BTAR reporter, similar to the Tat-Tap control. The largest group of positive clones encoded fusions to known RNA-binding proteins, most containing RGG repeats, and in general showed the highest activation levels (Table 1, Fig. 2). Several of the strongest and most specific clones were hnRNP proteins, including hnRNP A1 which was previously implicated in RNA export [35]. Sam68, which also showed good specificity in the Tat-hybrid assay, has been reported to enhance the activities of RRE- and CTE-dependent reporters and appears to work synergistically with Tap [36], [37]. Other identified RNA-binding proteins, such as FUS/TLS, EWS, and nucleolin, also have reported roles in export in addition to their activities in transcription, splicing, and ribosomal RNA transcription and biogenesis (reviewed in [38], [39]. Properties of the most active proteins are annotated in Table 1.

### Evidence for an interaction between the CTE, hnRNP A1, and Tap

We chose to focus follow-up experiments on hnRNP A1 for several reasons: 1) it was by far the dominant clone isolated from our library screen (three different clones found 21 times); 2) it showed high activation levels and strong specificity for the CTE; 3) it contains four RGG motifs, which is representative of many of the clones isolated; and 4) it has been implicated in RNA export and thus was a plausible candidate for a CTE-interacting protein.

We tested whether hnRNP A1 interacts directly with the CTE using RNA gel shift assays. A titration of GST hnRNP A1 alone (Fig. 3, lanes 2–6) showed a ladder of discrete hnRNP A1 species bound to the 185-nt CTE RNA, indicating multiple binding sites. Similar results were observed with an antisense version of the CTE (data not shown), suggesting that these complexes are relatively non-specific. In contrast, a titration of GST Tap showed the formation of one main complex at µM concentrations with the CTE RNA (Fig. 3, lanes 8–13), similar to previously reported results [19], [40]. Because the Tat-hybrid system can assemble larger multi-protein complexes with endogenous proteins *in vivo* [10], we reasoned that our observation of specificity in the reporter system might reflect the ability of Tat-fused hnRNP A1 to assemble more selectively on the CTE via an interaction with endogenous Tap. Indeed, a gel shift experiment using 2.5 µM Tap and titrating hnRNP A1 (Fig. 3, lanes 14–18) showed the formation of a supershifted band on the CTE RNA (lane 18), providing evidence that hnRNP A1 and Tap can assemble into a complex with the CTE RNA *in vitro*.

### Interacting domains of hnRNP A1

Given that hnRNP A1 is able to form a complex with CTE/Tap, we next evaluated a possible pairwise interaction between hnRNP A1 and Tap. hnRNP A1 is 320 amino acids long and contains two RRM domains (amino acids 1–184), an RGG domain (amino acids 195–234), and a C-terminal region that includes the M9 NLS/NES (amino acids 268–305) (Fig. 4A). GST-fused hnRNP A1, but not GST alone, was able to pull down *in vitro* translated [^35^S]-labeled Tap (full-length 1–619) (Fig. 4B, lanes 2 and 1). Similarly, GST-Tap but not GST pulled down *in vitro* translated hnRNP A1 (Fig. 4B lanes 4 and 3).

To assess which domains of hnRNP A1 mediate the interaction with Tap, we performed pull-down assays with GST-tagged Tap and a set of *in vitro* translated hnRNP A1 deletion proteins based on the protein domain structure and endpoints of hnRNP A1. A deletion protein containing the RGG domain (1–234) interacted as well as the full-length hnRNP A1 while the RRMs alone (1–194) did not interact with Tap (Fig. 4C, Lanes 4, 5, and 6). No binding was seen to GST alone. Although we were unable to express the RGG domain (195–234) alone using these methods, the overlap between 1–234 and 1–194 constructs suggested that the RGG domain (195–234) mediates the interaction with Tap.

Deletion analysis was also used to identify regions of hnRNP A1 important for CTE interactions in the Tat-hybrid context. Deletion constructs containing either the RRMs alone (1–194) or only the RGG and M9 domains (195–320) were engineered and tested in the CTE binding assay. Both constructs conferred moderate activity and specificity (Fig. 4D) suggesting that both the RRM and RGG/M9 domains of the protein contribute to the interaction between hnRNP A1 the CTE.

### Library clones were enriched for RGG domains

It was readily apparent that the list of highly activating clones was enriched for proteins that contained RGG domains, including four of the most active clones EWS, LSM14A/RAP55, nucleolin, and hnRNP R (Table 1). Intriguingly, we also identified 25 out-of-frame or non-coding clones that conferred activation of the CTE reporter and were enriched in Tat-fused RGG tri-peptide and/or RG di-peptide sequences (Table 2). The frequency of RGG sequences among ∼32,000 non-redundant human genes is 0.00027 per amino acid and was enriched about 30-fold in our screen. RG and RGG motifs often are observed in RNA-binding proteins (reviewed in [41], [42]) and are common sites of arginine methylation [24].

We tested the activity of three representative non-coding clones using the Tat-hybrid assay and these clones demonstrated modest activity and reasonable specificity for the CTE (Fig. 5). This result is consistent with the hypothesis that RGG domains are a determinant for complex formation on the CTE, as is apparently the case for hnRNP A1. In addition, several of the non-coding clones, as well as hnRNP A1, were substrates for arginine methylation *in vitro* (Fig. S1). While these modification data are provocative, they do not indicate whether methylation directly contributes to the CTE interaction.

## Discussion

Here we report a method to identify RNA-binding proteins from cDNA libraries using the Tat-hybrid system, which has been used previously to monitor RNA-protein interactions and to screen combinatorial RNA-binding peptide libraries [25]. The method complements others that have successfully identified new RNA-binding proteins, such as the commonly used three-hybrid system in yeast or more recent protein-immobilized microarrays [3]–[7]. A key advantage is that our screen is conducted in mammalian cells, allowing endogenous proteins to assemble into RNP complexes even though Tat may be fused to only one binding partner of a complex [10]. Our results suggest that we have identified new components of a multi-protein complex and, in this instance, the cellular context of the screen is likely to have been essential for achieving this result.

To evaluate the platform under actual screening conditions, we initially carried out a pilot screen using the cDNA library spiked with a positive control. Tat-Tap, present in the cDNA library at an initial concentration of 1∶100,000, was enriched 570-fold to 1∶175 after two rounds of library screening thus confirming that the platform was capable of detecting a rare, interacting clone. We next carried out the Tat-hybrid screen to identify potential CTE-interacting proteins. We found very substantial enrichment for proteins having RGG domains, including out-of-frame fusions randomly encoding RGG and RG sequences. The most abundant clone isolated was hnRNP A1, and *in vitro* gel shift and pull down experiments provide evidence that hnRNP A1 assembles on the CTE RNA in conjunction with Tap, a previously identified protein that specifically recognizes the CTE [19]. The interaction in part appears to be mediated by the RGG domains, which are known sites for arginine methylation [43]. Although RGG domains often are present in RNA-binding proteins, the roles of the modified arginines are not fully understood and may be important for RNA binding and/or protein-protein interactions [41], [42]. For example, the RGG box of Herpes Simplex Virus ICP27 is used to bind RNA and is important for Tap-mediated export [44] while methylation of arginines in Aly/Ref reduces its RNA-binding affinity to allow the RNA to be handed off to Tap [45]. The interplay between RNA-binding affinity and protein-protein interactions during the assembly/disassembly of RNPs is consistent with a role for the RRM and RGG domains of hnRNP A1 in the Tap-mediated CTE pathway, as suggested by our data.

Previous studies have shown that Tap has a weak affinity for pre-mRNA and is recruited through adaptor proteins of the Aly/Ref/Yra1 family [46], [47]. However Aly/Ref is dispensable for bulk mRNA export, suggesting that its role in recruiting Tap can be substituted with other factors [48]. Tap recruitment to CTE-containing RNA is unique because of its high affinity for the CTE and because CTE-containing RNAs bypass several steps in RNA processing, resulting in an RNP composition different than spliced mRNAs and not, for example, containing Aly/Ref [49]. We speculate that hnRNP A1 and perhaps other proteins identified in our screen may bind the CTE and serve as adaptor proteins for Tap. hnRNP proteins in general are relatively non-specific RNA-binding proteins, however, it does not necessarily mean that this family of proteins cannot be influenced by other specifically bound proteins or are not key players in functional complexes. A possible role of the RGG domains in mediating interactions with Tap is consistent with the observation that Yra1 in yeast is required to recruit the Tap homolog Mex67 [50] and that the SR proteins can act as Tap adaptors [51]. Interestingly, the yeast SR-like protein Npl3 may serve as an adaptor for Mex67 [52] and, despite some functional differences, often is regarded as the yeast homolog of hnRNP A1. The diversity of proteins that coat mRNAs and their range of binding specificities, particularly hnRNP proteins [53], suggest the existence of complex and combinatorial export pathways that can be exploited by viruses such as MPMV.

## Supporting Information

Figure S1 ***In vitro*** **methylation of hnRNP A1 and non-coding clones.** GST fusions to selected non-coding peptides were constructed and *in vitro* methylation assays were performed with recombinant Prmt1 methylase and [^3^H]-Ado-Met [31]. (Top) *In vitro* methylation assays using N3, N12, and N26 GST-fusion proteins and purified Prmt1 methylase, with GST-hnRNP A1 as a positive control and GST alone as a negative control. All three peptides, as well as the HnRNP A1 control, were substrates for methylation (lanes 7–10). Interestingly, the N3 peptide that contained eight RG sequences and displayed the highest level of methylation also conferred the strongest binding activity of this class in the Tat-hybrid assay. No protein was methylated in the absence of enzyme (lanes 1–5). (Bottom) Expression levels of each input protein in the methylation reaction were determined by running a duplicate gel and staining with Coomassie blue.
(TIF)
