# Supplementary figures and images for: A Cell-Based Method for Screening RNA-Protein Interactions: Identification of Constitutive Transport Element-Interacting Proteins
Source: PLoS One. 2012 Oct 25;7(10):e48194. doi: 10.1371/journal.pone.0048194 (PMC3485056; doi:10.1371/journal.pone.0048194)

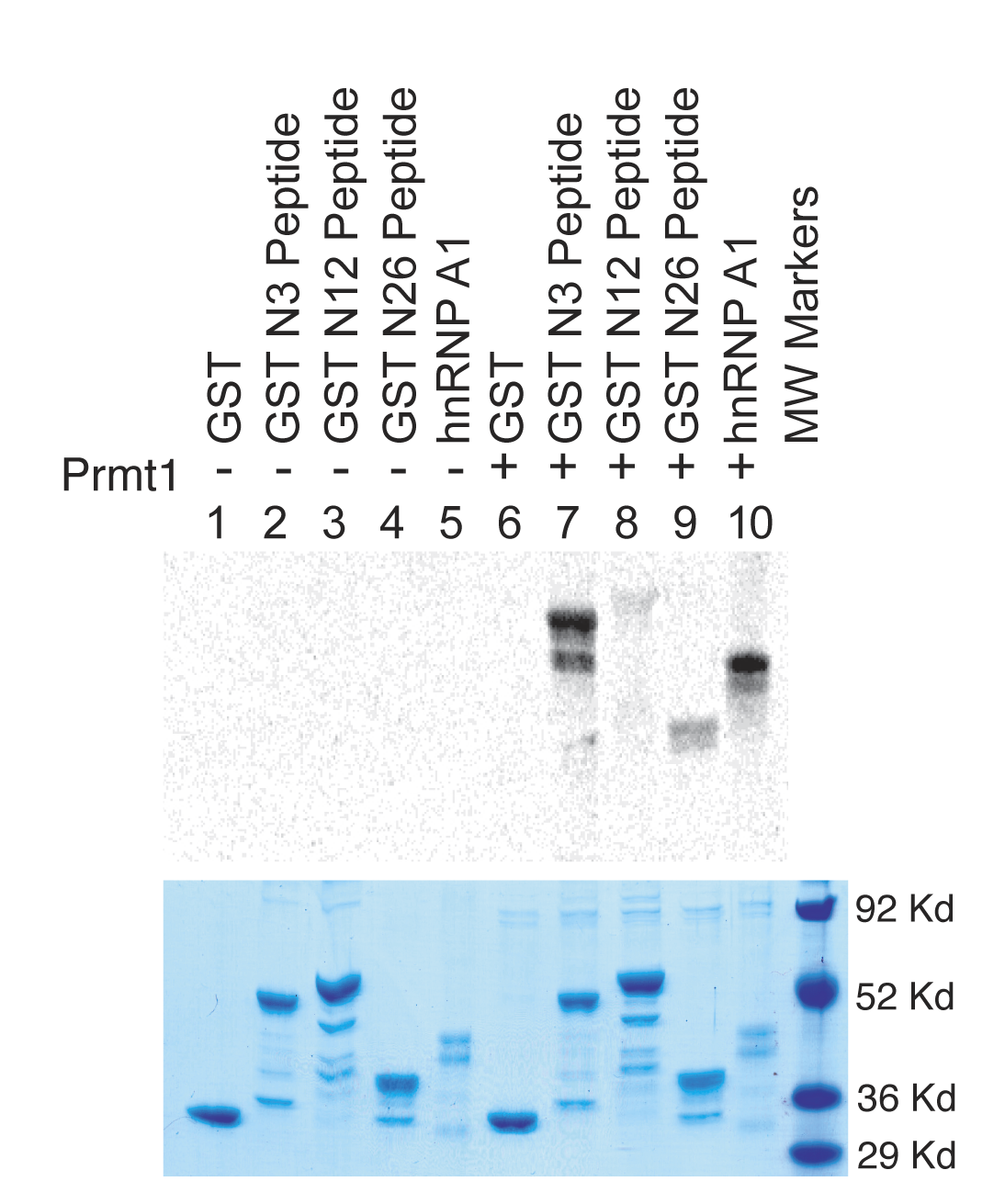

Supplement: Figure S1 — In vitro methylation of hnRNP A1 and non-coding clones. GST fusions to selected non-coding peptides were constructed and in vitro methylation assays were performed with recombinant Prmt1 methylase and [3H]-Ado-Met [31]. (Top) In vitro methylation assays using N3, N12, and N26 GST-fusion proteins and purified Prmt1 methylase, with GST-hnRNP A1 as a positive control and GST alone as a negative control. All three peptides, as well as the HnRNP A1 control, were substrates for methylation (lanes 7–10). Interestingly, the N3 peptide that contained eight RG sequences and displayed the highest level of methylation also conferred the strongest binding activity of this class in the Tat-hybrid assay. No protein was methylated in the absence of enzyme (lanes 1–5). (Bottom) Expression levels of each input protein in the methylation reaction were determined by running a duplicate gel and staining with Coomassie blue. (TIF) [file pone.0048194.s001.tif]
